# Correction: Alfat et al. Phase Field Models for Thermal Fracturing and Their Variational Structures. *Materials* 2022, *15*, 2571

**DOI:** 10.3390/ma15103623

**Published:** 2022-05-19

**Authors:** Sayahdin Alfat, Masato Kimura, Alifian Mahardhika Maulana

**Affiliations:** 1 Physics Education Department, Halu Oleo University, Kendari 93232, Southeast Sulawesi, Indonesia; 2 Division of Mathematical and Physical Sciences, Graduate School on Natural Science and Technology, Kanazawa University, Kakuma, Kanazawa 920-1192, Japan; fiansmamda@gmail.com; 3 Faculty of Mathematics and Physics, Kanazawa University, Kakuma, Kanazawa 920-1192, Japan; mkimura@se.kanazawa-u.ac.jp

## Error in Equations

The authors were not aware of typographical errors made in the writing phase, and, hence, wish to make the following corrections to the original paper [1]. We apologize for these mistakes. There are four typo mistakes in the original paper, which are corrected as follows:

- Equation (1) is corrected as:
  divσ[u]=β∇Θ  in Ω×[0,T],
- The following equation
  −μΔu−(λ+μ)∇(divu)= β∇Θ.
  is corrected as:
  μΔu+(λ+μ)∇(divu)=β∇Θ.
- Equation (19a) is corrected as:
  $$\tilde{\mathrm{div}}\tilde{\sigma }\left[\tilde{u}\right]=\tilde{\nabla }\tilde{\Theta }\;\;\mathrm{in}\;\tilde{\Omega }\times \left[0,\tilde{T}\right],$$
- Equation (39a) is corrected as:
  $$\mathrm{div}\left({\left(1-z\right)}^{2}\sigma \left[u\right]\right)={\left(1-z\right)}^{2}\nabla \Theta \;\;\mathrm{in}\;\Omega \times \left[0,T\right],$$
  The original article has been corrected.
